# Bacteriophage MS2 displays unreported capsid variability assembling *T* = 4 and mixed capsids

**DOI:** 10.1111/mmi.14406

**Published:** 2019-11-05

**Authors:** Natàlia de Martín Garrido, Michael A. Crone, Kailash Ramlaul, Paul A. Simpson, Paul S. Freemont, Christopher H. S. Aylett

**Affiliations:** ^1^ Section of Structural and Synthetic Biology Department of Infectious Disease Imperial College London London SW7 2AZ UK; ^2^ UK DRI Care Research and Technology Centre Imperial College London London UK; ^3^ Centre for Structural Biology Department of Life Sciences Imperial College London London SW7 2AZ UK; ^4^ London BioFoundry Imperial College Translation & Innovation Hub White City Campus, 80 Wood Lane London W12 0BZ UK

## Abstract

Bacteriophage MS2 is a positive‐sense, single‐stranded RNA virus encapsulated in an asymmetric *T* = 3 pseudo‐icosahedral capsid. It infects *Escherichia coli* through the F‐pilus, in which it binds through a maturation protein incorporated into its capsid. Cryogenic electron microscopy has previously shown that its genome is highly ordered within virions, and that it regulates the assembly process of the capsid. In this study, we have assembled recombinant MS2 capsids with non‐genomic RNA containing the capsid incorporation sequence, and investigated the structures formed, revealing that *T* = 3, *T* = 4 and mixed capsids between these two triangulation numbers are generated, and resolving structures of *T* = 3 and *T* = 4 capsids to 4 Å and 6 Å respectively. We conclude that the basic MS2 capsid can form a mix of *T = *3 and *T = *4 structures, supporting a role for the ordered genome in favouring the formation of functional *T = *3 virions.

## Graphical abstract

- MS2 packed with non‐genomic RNA displays capsid variability and produces *T* = 3 and small percentages of *T* = 4 and mixed *T* value virions.
- We present cryo‐EM structures of *T* = 3 and *T* = 4 icosahedral MS2 capsids.
- The *T* = 4 icosahedral architecture does not form the standard maturation protein‐binding site.

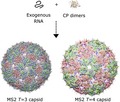

**Abbreviations**
CP: coat protein
IAU: icosahedral asymmetric unit
MP: maturation protein
VLP: virus‐like particle

## Introduction

Viruses are highly variable macromolecular assemblies formed by a protein capsid which surrounds a genome composed of either single‐ or double‐stranded DNA or RNA (Prasad and Schmid, 2012). Regardless of their shape, geometry or size, all viruses require a host organism to replicate. As such, all viruses have evolved capsid structures which are evolutionarily constrained to perform two main functions: to ensure the packaging and therefore protection of their genome; and to enable host interactions required for infectivity. Viral capsid assembly occurs via different mechanisms dependent on the type of genome: while double‐stranded DNA (dsDNA) viruses normally pack their genome into a preformed capsid (Bazinet and King, 1985), viruses containing single‐stranded RNA (ssRNA) assemble their capsids around their genome (Klug, 1999; Sun *et al.*, 2010; Stockley *et al.*, 2013).

The size of a viral genome is restricted by the fact that it must be encapsulated sufficiently by a capsid of limited complexity, as the genome must encode structural capsid proteins as well as functional proteins. Therefore, to minimise the amount of genetic information required, capsids are typically built from multiple copies of a few proteins and are usually highly symmetric (Crick and Watson, 1956). Icosahedral geometry is very common in spherical viruses because it allows the placement of 60 identical subunits with equivalent contacts between them. However, it has been observed that most spherical viruses form their capsids with multiples of 60 subunits, while maintaining icosahedral symmetry. To achieve this, the triangular face of the icosahedron must be enlarged and divided into smaller triangles, in a process described as triangulation (Caspar and Klug, 1962). Therefore, icosahedral viruses can be described by a triangulation number, *T*, which defines the number of distinct subunit conformations forming the icosahedral asymmetric unit (IAU). These conformations are considered quasi‐equivalent between them. This high level of symmetry can enforce strict geometric restraints during virus capsid assembly, thus leading to the production of a homogeneous pool of viral particles.

Bacteriophage MS2 is a ssRNA virus containing a 3 569 nucleotide long genome (Fiers *et al.*, 1970) confined in a capsid with one maturation protein monomer and 89 coat protein dimers. MS2 infects *E. coli* by interacting with its F‐pilus through the maturation protein (Valentine and Strand, 1965; Brinton, Gemski, and Carnahan, 2006). This interaction allows the phage to deliver its genome and the MP inside the host cell, while the capsid shell is emptied and left outside the bacterium (Stockley *et al.*, 2016). Therefore, viral particles lacking MP lose infectivity and do not bind to *E. coli* F‐pili (Krahn *et al.*, 1972; Roberts and Steitz, 2006). MS2 particles *in vivo* have capsids with *T* = 3 pseudo‐icosahedral symmetry (Valegard *et al.*, 1990; Golmohammadi *et al.*, 1993) that are co‐assembled around the genome. The ssRNA forms secondary structural elements that directly interact with the coat proteins acting as packaging signals, with the 19‐nt long stem loop being the best characterised (Stockley *et al.*, 2016). During the assembly process, the RNA specifies the three quasi‐equivalent conformations of the coat protein that form the IAU, named A, B and C (Stockley *et al.*, 2007). These subunits interact forming two different types of dimers: an asymmetric A/B extended from the fivefold axes to the threefold axes and a symmetric C/C dimer sitting on the twofold axes. Therefore, the capsid can be considered a construction of 90 dimers (Roberts and Steitz, 2006). During the nucleation process, a single copy of the maturation protein is included in the MS2 capsid replacing one C/C dimer, and thereby breaking the symmetry of the capsid and resulting in small structural changes in the surrounding coat proteins (Dent *et al.*, 2013; Koning *et al.*, 2016; Dai *et al.*, 2017).

Recent studies revealed the structure of the MS2 ssRNA genome assembled within its capsid (Dent *et al.*, 2013; Koning *et al.*, 2016; Dai *et al.*, 2017), demonstrating that the formation of the physiological *T* = 3 capsid is specifically guided by the interaction between stem‐loops in the folded genome and the maturation protein. The authors surmised that the formation of non‐standard capsids had been strongly selected against, resulting in the observed ordered genome. However, as such non‐standard capsids had not been observed for packed virions, they were unable to provide a definitive explanation for this selective pressure. Here, we report two cryo‐EM structures of the MS2 capsid: the previously reported *T* = 3 symmetry form; as well as a novel *T* = 4 icosahedral symmetry variant, both loaded with a 155 bp sequence containing the MS2 capsid interacting stem‐loop. Our results support the notion that MS2 exhibits structural variability, in the absence of the highly ordered viral genome, accommodated by slight structural changes in coat proteins (Dai *et al.*, 2017). Importantly, analysis of the non‐standard *T* = 4 variant structure shows that this capsid architecture changes the environment of the maturation protein‐binding site, thus making less plausible its inclusion into the capsid without major structural changes. The lack of maturation protein would result in non‐infectious particles and would explain the requirement for the discrimination towards the *T* = 3 setting through regulation of the capsid assembly process by the highly ordered MS2 genome.

## Results

### Generation of MS2 virus‐like particles

MS2 has been widely used as a virus‐like particle for the packaging of heterologous RNA as an internal standard for RT‐PCR (Pasloske *et al.*, 1998; Zhan *et al.*, 2009). Although particles have been made available commercially for diseases like Hepatitis C and Enterovirus (Armoured RNA®), the sequences of the packaged RNA are not publicly available. Various particle purification methodologies have been suggested and single vectors have been described that allow for the purification of MS2 virus‐like particles with user‐specified RNA sequences using affinity chromatography (Mastico *et al.*, 1993; Mikel *et al.*, 2017). We therefore created a single plasmid vector with the CP‐His‐CP MS2 dimer (Peabody and Lim, 1996; Mikel *et al.*, 2017) which was co‐expressed with maturation protein and a 155 bp heterologous RNA sequence incorporating a modified MS2 stem‐loop (c‐variant *pac site*) (Wei *et al.*, 2008) (Supplementary Figs 1A–C, and 2A). We confirmed that this specific RNA was packaged by electrophoretic mobility shift assay and RT‐PCR (Supplementary Fig. 2B and C).

### Observation of variable capsids for both CP‐His‐CP and wt‐CP MS2 capsid particles

We prepared negatively‐stained EM grids using freshly purified virus‐like particles generated with the CP‐His‐CP MS2 dimer. Micrographs revealed a large number of intact capsids, implying that virion assembly had taken place robustly (Supplementary Fig. 3A). Detailed inspection of the micrographs showed that there was substantial heterogeneity in particle size, prompting us to pursue more detailed structural analysis using cryo‐EM. There were two predominant populations: ‘small’ particles (~85%) with an approximate diameter of ~280 Å and ‘large’ particles (~15%) with a maximal diameter of ~330 Å, some of which were slightly non‐spherical or elongated (~1%) (Supplementary Fig. 3A–C). The two sets of icosahedral capsids were analysed separately, while mixed capsids were too variable to process in three‐dimensions. The particle size difference was substantial but not sufficient for clear biomechanical separation of the two populations as they also possess similar shapes.

On the observation of divergent symmetry VLPs, we created an otherwise identical wild‐type (wt) construct with monomeric CP and prepared cryo‐EM grids to confirm or deny that the presence of the CP‐His‐CP dimer was not solely responsible for the formation of mixed and large capsids as previously reported for non‐wild‐type constructs (Peabody and Chakerian, 1999; Plevka *et al.*, 2008, 2009; Asensio *et al.*, 2016; Zhao *et al.*, 2019). We confirmed similar observations in the micrographs of the wild‐type setting; in the higher molecular weight fractions of the wt‐CP sample, the majority of the particles belonged to the smaller class, ~85% of the total on sorting by 2D classification, while several varieties of ‘large’ particles accounted for the remaining ~15%, of which ~6% later proved to be large icosahedral particles (Supplementary Fig. 3D). Although, in the wt‐CP, it is clear that mixed, rather than icosahedral, capsids were the predominant non‐standard form, these results demonstrate that ‘large’ and ‘mixed’ VLPs were indeed formed during the assembly of MS2 capsids by wt‐CPs with the RNA tested. We note that the wt‐CP large and mixed icosahedral particles were less well‐ordered than those identified from the dimeric CP, but conclude that the architectures of these non‐standard capsids are clearly relevant to wt‐CP VLP formation in the presence of non‐genomic RNA.

### Three‐dimensional structural characterisation of CP‐His‐CP MS2 VLPs

**Figure 1:**
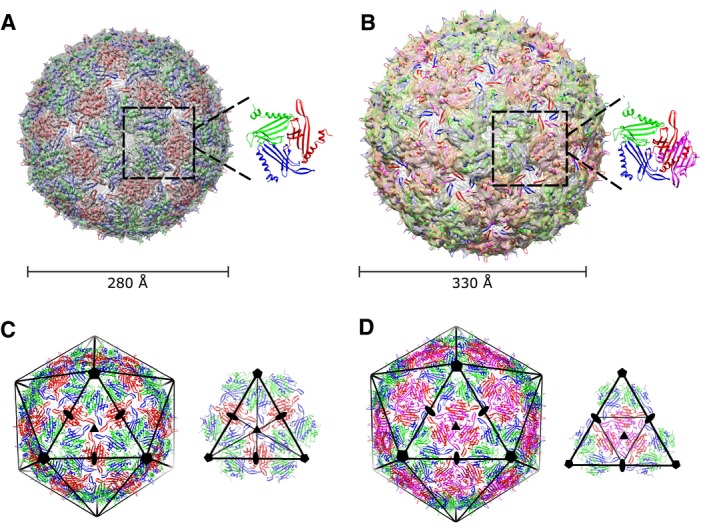
Structural analysis of the *T* = 3 and *T* = 4 architecture of the MS2 capsid. A. The reconstructed density obtained for the *T* = 3 capsid at 4 Å is shown as a transparent surface in grey. The corresponding model cartoon was fitted on the density map. The IAU is shown on the right with each CP quasi‐equivalent conformation depicted in a different colour: A in blue, B in green and C in red. B. The reconstructed density obtained for the *T* = 4 capsid at 6 Å is shown as a transparent surface in light yellow with the corresponding model cartoon fitted on the map. The IAU is shown on the right. Each CP quasi‐equivalent conformation is shown in a different colour as in Fig. 1A with the fourth chain, D, depicted in magenta. C. On the left, *T* = 3 capsid structure with an icosahedral volume and its symmetries superimposed. On the right, the icosahedral triangular face of the *T* = 3 capsid with the corresponding symmetries is shown. D. On the left, the *T = *4 capsid structure in cartoon representation with an icosahedral volume and its symmetries superimposed. On the right, the icosahedral triangular face of the *T = *4 with the corresponding symmetries is shown. All views are from the outside of the capsid.

Single‐particle analysis of the smaller MS2 capsid particles produced a reconstruction of the *T = *3 icosahedral MS2 architecture (Fig. 1A), consistent with previously described MS2 structures (Valegard *et al.*, 1990; Golmohammadi *et al.*, 1993). Reconstruction of the ‘larger’ particles resulted in a reconstruction of a *T* = 4 icosahedral geometry previously unreported for MS2 (Fig. 1B). The heterogeneous particles appeared to exhibit a mix of partial *T* = 3‐like and *T* = 4‐like symmetry, probably *T* = 3, *Q* = 4 symmetry (Prasad and Schmid, 2012), based on their variation in diameter between the two extremes corresponding to these two settings (Supplementary Fig. 3A–D).

The maturation protein was co‐expressed with the coat protein in these samples, and its expression confirmed by mass‐spectrometry (Supplementary Table 1). However, MP was not visible in our VLPs due to the absence of MP interacting stem‐loops on the packaged RNA. For completeness, we confirmed that capsid variability remained present when coat protein dimers were expressed in the absence of the maturation protein. Using negatively‐stained EM grids, we were able to consistently detect both capsid symmetries, *T* = 3 and *T* = 4, as well as mixed capsids, based on their size and two‐dimensional classification (Supplementary Fig. 3E).

### Overall structure of the *T* = 4 MS2 CP‐His‐CP capsid

Cryo‐EM data were collected for an identical sample on an F20 equipped with an early Falcon detector. Single particle analysis of the large MS2 CP‐His‐CP particles revealed that they represented a *T* = 4 icosahedral architecture in comparison to the previous *T* = 3 structures. Conformational heterogeneity and the collection system restricted the resolution to 6 Å (*T* = 3 reached 4 Å for comparison – which is the highest resolution reconstruction achieved on this instrument to date), which prevented high‐resolution analysis of the detailed interactions between CPs, but allowed clear and unambiguous placement of the known CP structure. Resolution was estimated by gold‐standard FSC = 0.143 (Supplementary Fig. 4), and the individual coat protein structure (PDBID: 5TC1) was fitted into the density, with each coat protein dimer being placed as an independent rigid body (Fig. 1A and B). These larger particles exhibited a diameter of 330 Å and would be formed by 240 coat proteins (Fig. 1B). Each IAU is formed by four asymmetric quasi‐equivalent conformations of the coat protein, designated A, B, C and D, in comparison to the asymmetric subunit of the physiological *T* = 3 structure composed of three quasi‐equivalent conformations (Fig. 1A and B). The addition of the fourth chain to the basic icosahedral building block provides the explanation for the increased *T* = 4 capsid size.

### Dimer organisation within the *T* = 4 capsid

In the *T* = 3 MS2 structure, the building blocks of the capsid are two different types of coat protein dimers: an asymmetric A/B dimer extending from the fivefold axes to the threefold axes and a symmetric C/C dimer sitting on the icosahedron twofold axes. The fivefold vertices are surrounded by A/B dimers forming a ring with the FG‐loops of chain B and the threefold symmetry is surrounded by six alternating A/B and C/C dimers (Fig. 1C). The addition of a fourth subunit in the *T* = 4 capsids introduces a series of limited changes in the organisation of the coat protein dimers. The A/B dimers are conserved in an identical position to that in the *T* = 3 setting. However, this is not the case for the C/C dimers, which no longer exist in the *T* = 4 form. The larger *T* = 4 capsids contain a new environment for a dimer comprising chains C and D in our structure; three C/D dimers sit on the centre of the icosahedral triangular face around the threefold symmetry origin, establishing a trimeric interaction through their edges (Fig. 1D). Therefore, while in the *T *= 3 setting, the threefold symmetry was formed by IAU of chains A, B and C, in *T *= 4 capsids, the 3‐fold symmetry is formed by IAU and this novel trimeric interaction between C chains. These C/D dimers interdigitate their FG‐loops with the A/B ones, creating a pseudo sixfold symmetry origin on the sides of each triangular face, while conserving the icosahedral twofold symmetry (Fig. 1D). The result of this change of setting is that there are no longer any symmetric C/C dimers linking the fivefold symmetric capsid rings: the only linkage between such rings is through threefold C/D dimer contacts.

### Structural comparison of the threefold symmetry of *T* = 3 and *T* = 4 icosahedral capsids

**Figure 2:**
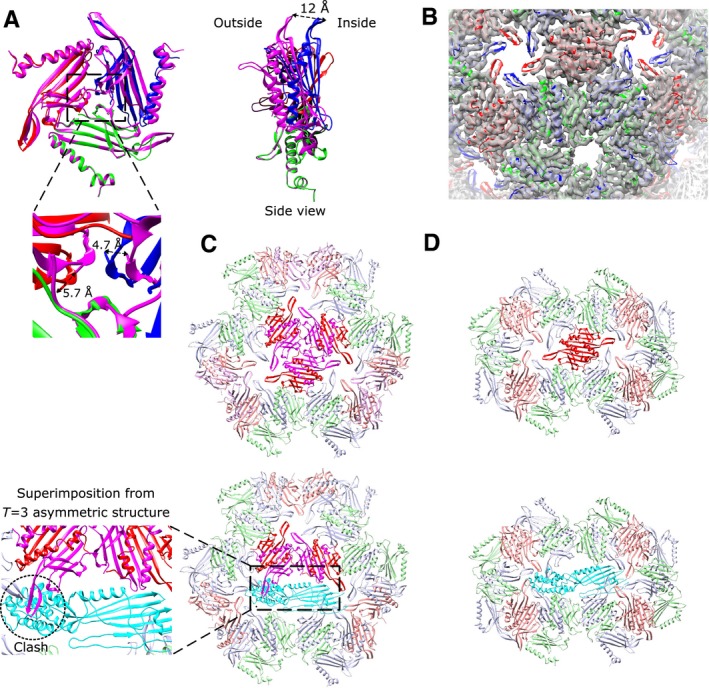
A threefold interaction replaces the maturation protein incorporation site in *T = *4 capsids. A. Superimposition of the *T* = 3 pseudo threefold symmetry and the *T* = 4 threefold symmetry viewed from the outside of the capsid. Different conformations of coat proteins are depicted in different colours with chain A in blue, B in green, C in red and D, from *T* = 4, in magenta. Below, closer view of the threefold symmetry centre. Distances between atoms are depicted in black‐dashed arrows. B. Cartoon representation of the *T* = 3 threefold symmetry fitted in the high‐resolution *T* = 3 density map section. C. The incorporation of a fourth chain in the IAU subunit changes the dimer composition of the MS2 capsid and a triple interaction between C/D dimers replaces the *T* = 3 C/C dimers. The second panel and inset show a superimposition of the MP from the asymmetric *T* = 3 structure for the purposes of comparison (PDB ID: 5TC1) and its potential clash with the adjacent C/D dimer on the threefold. D. Binding of the MP replacing one C/C dimer in the *T* = 3 MS2 capsid. The MP is shown in cartoon representation depicted in cyan blue in each case. All views are from the outside of the capsid, unless otherwise stated.

The change in setting between *T* = 3 and *T* = 4 leaves the greater part of the MS2 capsid structure as it is in the *T = *3 situation: the points of variation are almost entirely constrained to the additional, D, capsid protein conformation. The C/D dimers surround the threefold symmetry origin at the centre of the icosahedral face (Fig. 1D), a contact which replaces the C/C dimer on the twofold symmetry in the *T* = 3 setting. This new threefold interaction is extremely similar to that formed by the IAU in the physiological *T* = 3 architecture, explaining the structural plasticity that allows the coat protein to form both architectures. However, whereas in the *T = *3 capsid, the pseudo threefold symmetry is formed by three different coat protein conformers, A, B and C, the new threefold symmetry origin in the *T = *4 capsid setting comprises only one conformer: D (Figs 1C, D and 2B).

The accommodation of this extra subunit in the larger capsid architecture results in two slight changes in the threefold symmetry origin of the *T = *4, in comparison to the pseudo threefold symmetry formed by the IAU in the *T = *3 architecture. There is a slight distortion inward and a clockwise rotation of the D chains at the centre of the threefold symmetry, which causes a ~12 Å displacement of the FG‐loops outwards by the edge of the next dimer, enlarging the whole capsid accordingly (Fig. 2A). These rotations and movements create a slightly ‘buckled’ region in the surface of the *T* = 4 capsid, which we would expect to make the structure less favoured in comparison to the physiological *T* = 3 form.

### *T* = 4 MS2 capsids do not form the standard maturation protein incorporation site

Incorporation of the maturation protein into the *T* = 3 capsid is essential for the construction of functional viral particles able to infect *E. coli* (Dent *et al.*, 2013). During the infection process, the maturation protein must be accessible on the surface of the virus to bind to the F‐pilus of the host bacteria (Curtiss and Krueger, 1974). A single copy of the maturation protein both serves as the attachment point to the bacterial receptor, and guides both the RNA and the MP inside the host cell, with the coat proteins remaining outside. To achieve this, in the physiological *T* = 3 MS2 capsids, the maturation protein replaces a C/C coat protein dimer at the twofold symmetry axis during the capsid assembly process, inducing small structural changes in the surrounding coat proteins and disrupting the capsid symmetry (Dent *et al.*, 2013; Koning *et al.*, 2016; Dai *et al.*, 2017; Fig. 2D). In the *T* = 4 MS2 architecture, we observed in this study, the coat protein dimers' organisation around the icosahedral capsid is different from that in the physiologically selected capsids. The incorporation of an additional chain in the IAU (Fig. 1B) not only prevents the formation of C/C dimers but also allows a triple interaction of C/D dimers arranged in a threefold symmetric fashion at the centre of the icosahedral face (Fig. 1D). Consequently, the new C/D trimer is noticeably rotated with respect to the standard *T* = 3 setting (Fig. 2A), and results in a theoretical clash between the adjacent C/D dimer and a postulated maturation protein (superimposed from the *T* = 3 structure, PDB ID 5TC1) replacing a given C/D dimer (Fig. 2C).

## Discussion

As a spherical ssRNA virus, bacteriophage MS2 co‐assembles its capsid around its genome (Stockley *et al.*, 2016), forming a *T* = 3 icosahedral capsid containing 90 coat protein dimers (Valegard *et al.*, 1990; Golmohammadi *et al.*, 1993). This conformation allows the binding of the maturation protein during the assembly process, which replaces one of these coat protein dimers on a twofold axis (leaving 89), and breaks the capsid symmetry (Dent *et al.*, 2013; Koning *et al.*, 2016; Dai *et al.*, 2017). During infection, this maturation protein interacts with the F‐pilus of *E. coli* to introduce the viral genome into a new host (Valentine and Strand, 1965). The genomic RNA is highly ordered (Toropova *et al.*, 2008; Dykeman *et al.*, 2010) and specifies the three quasi‐equivalent conformations of the coat protein to form dimers, and the contacts to the maturation protein during the virion assembly (Stockley *et al.*, 2007; Dykeman *et al.*, 2010; Rolfsson *et al.*, 2010). Hence, the genome of MS2 is thought to restrict the folding pathway to select for the assembly of physiological *T* = 3 capsids, preventing the formation of other capsid forms (Dai *et al.*, 2017). Previous studies identified both smaller and larger non‐infectious MS2 particles formed *in vitro* (Sugiyama *et al.*, 1967), however, the architecture of these non‐standard capsids (mostly likely mixed *T = *1*, T = *3 and *T = *3*, T = *4 structures given our results and those of Asensio *et al.*, 2016) and why they might be problematic for infection remained unknown.

Our cryogenic electron microscopy analysis of virus‐like MS2 particles has revealed substantial capsid variability when the virus is assembled with an exogenous RNA sequence which does not incorporate any stem‐loop structure known to interact with the maturation protein but does contain the MS2 packaging signal (Supplementary Fig. 2A–C, Supplementary Table 3) (Legendre and Fastrez, 2005; Wei *et al.*, 2008). Single particle analysis revealed that, in these conditions, MS2 phage could assemble its capsid in both *T* = 3 and *T* = 4 icosahedral settings, as well as poorly defined hybrids of these two architectures. The formation of icosahedral capsids with different triangulation numbers has been previously reported for other viruses (Venkatakrishnan and Zlotnick, 2016; Jung *et al.*, 2019), which is not wholly unexpected considering the close symmetry relationships between related icosahedral triangulations (Prasad and Schmid, 2012).

We have resolved the architecture of MS2 VLPs in a *T* = 4 setting to 6 Å resolution with a Tecnai F20 (FEI) equipped with an early Falcon camera, revealing that the formation of *T* = 4 capsids is sustained by MS2 coat proteins. The symmetry enforced by this novel MS2 architecture changes the dimer organisation, forming a new trimeric interaction between C/D dimers in the *T = *4 capsids that are rotated and expanded with respect to the standard *T* = 3 setting, thereby being less favourable in terms of buried interaction surface. An important consequence is the removal of the C/C dimer on the twofold symmetry axis where the maturation protein (responsible for interacting with the bacterial receptor and infecting the host) is incorporated during the standard capsid assembly process. It is clear that incorporation of the maturation protein in place of one of the new C/D dimers would require substantial conformational changes in the surrounding coat protein network, relative to the situation in the *T* = 3 capsid, to avoid clashes with nearby FG loops (Fig. 2C/inset).

**Figure 3:**
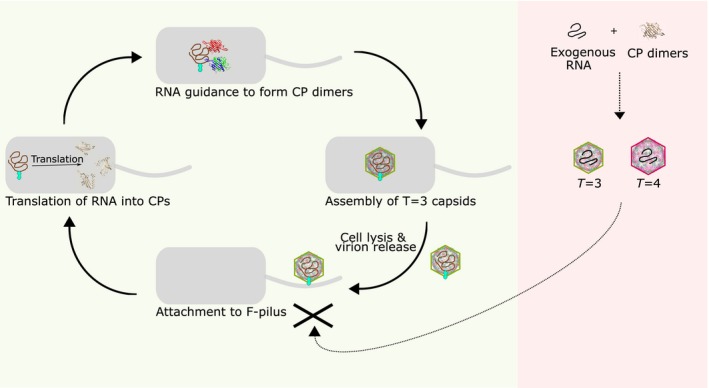
Bacteriophage MS2 lifecycle. On the left, MS2 lifecycle: the stem‐loops in the folded genome interact with the maturation protein to guide the packaging of the coat proteins into *T* = 3 capsids. After capsid assembly, the *E.coli* infected cell is lysate and virions are released to infect new cells. A single copy of the MP both serves as the attachment point to the initial bacterial receptor, the F‐pilus, and to guide the RNA into the host cell. Only the RNA and the MP get into the target cell with the coat protein capsid remaining outside. Once inside, the genome expresses CP proteins that are assembled into new capsids and the cycle starts again. When the RNA is packed with an incorrect RNA (right panel) containing the MS2 capsid packaging signal but missing the MP interacting loop, the capsid can be assembled into either *T* = 3 or *T* = 4 capsids, but will be non‐infectious.

There is evidence to suggest that such large conformational changes can be tolerated within capsids: the incorporation of the MP into MS2, and similar virions, has been reported to cause conformational changes in neighbouring coat proteins, and to weaken their interactions (Gorzelnik *et al.*, 2016; Dai *et al.*, 2017), while the MS2 MP is known to exhibit substantial orientational variation and flexibility when binding to the F‐Pilus (Meng *et al.*, 2019) It is not possible, therefore, for us to simply conclude from our observations that a proportion of *T* = 4 virions do not incorporate the MP. The question is how frequently the comparatively unstable *T* = 4 setting would tolerate such further destabilisation; we can note that larger MS2 particles were previously found to be essentially non‐infectious (Sugiyama *et al.*, 1967). Overall, our results support the notion that one role of the ordered MS2 genome, which regulates capsid assembly, is to disfavour unstable, non‐functional *T* = 4 capsids, and favour stable and infectious *T* = 3 virions, thus avoiding an unnecessary waste of coat protein building blocks (Fig. 3).

In the absence of RNA, MS2 CP dimers have been previously observed to form small octahedral particles which are non‐infectious (Plevka *et al.*, 2008), and as a consequence of its large interior volume and its ability to form non‐infectious capsids, MS2 can be loaded with a variety of cargos using different approaches (Wu *et al.*, 2005; Ashley *et al.*, 2011). Given this property, MS2 is already a focus of research in the field of therapeutic RNAs and as an internal standard for RT‐PCR disease detection, where several successful attempts have utilised MS2 as an RNA carrier to protect nucleotides from RNase degradation (Pasloske *et al.*, 1998; Uhlenbeck, 1998). However, with the advent of RNA‐guided nuclease mediated gene editing, there is growing interest in the delivery of RNA for transient gene expression. Current viral vectors already have packaging limitations which place severe restrictions on their use (Ran *et al.*, 2015) and thus the small size of the MS2 genome (Fiers *et al.*, 1970) may have precluded further investigation. Therefore, our work, along with previous work that has described the use of MS2‐chimeric retrovirus‐like particles to overcome this obstacle (Li *et al.*, 2014; Knopp *et al.*, 2018) and evidence of large cargos being incorporated into MS2 in the past (Zhan *et al.*, 2009; Zhang *et al.*, 2015), makes engineering MS2 an attractive prospect for future research.

## Experimental procedures

### MS2 construct cloning

Three MS2 virus‐like particle expression constructs were cloned: the MS2 maturation protein and a CP‐His‐CP dimer; the maturation protein and the wild‐type coat protein; and finally, the wild‐type coat protein without maturation protein. Additional information on how these constructs were made can be found in the Supplementary Methods. All constructs have an 155bp HIV *gag* nucleic acid sequence (Supplementary Table 3) with c‐variant *pac site* (Wei *et al.*, 2008) (Supplementary Fig. 2A). To exclude the possibility of dimer contamination of the monomeric sample, a single colony was extracted, verified by sequencing and EM analysis performed in parallel (Supplementary Fig. 3D).

### MS2 production and purification

All phage constructs were expressed in Rosetta2™ (DE3) pLysS cells (Merck). A starter culture was grown overnight at 30°C in a shaking incubator (180 rpm) by inoculating a single colony in 5 ml of Terrific Broth (Merck) supplemented with kanamycin (50 µg ml^−1^) and chloramphenicol (35 µg ml^−1^). A 200 ml culture of Terrific Broth, supplemented with kanamycin (50 µg ml^−1^) and chloramphenicol (35 µg ml^−1^), was then inoculated with 0.8 ml of overnight culture and grown to an OD of 0.6–0.8 at 30°C in a shaking incubator (180 rpm). The culture was then induced with 1 mM IPTG and left to grow overnight.

Cells were harvested by centrifugation at 3220× *g* at 4°C, followed by resuspension in 4 ml of sonication buffer (50 mM Tris‐HCl pH 8.0, 5 mM MgCl_2_, 5 mM CaCl_2_, 100 mM NaCl) described previously (Mikel *et al.*, 2017) and supplemented with 700U RNAse A (Qiagen), 2500U BaseMuncher (Expedeon) and 200U Turbo DNAse (Thermo Fisher Scientific). The cells were then lysed by sonication (50% amplitude, 30 seconds on, 30 seconds off, 4 pulses) at 4°C. Lysates were incubated at 37°C for 90 minutes to eliminate all contaminating nucleic acids. The incubated sample was centrifuged at 16,602× *g* at 4°C for 15 minutes and the supernatant filtered with a 5 µm CA filter (Minisart NML, Sartorius).

For the wild‐type MS2 construct, the filtered supernatant was applied to a glycerol gradient from 10 to 40% 160,000× *g* (30 000 rpm) 20 h in a SW40 rotor. For CP‐His‐CP MS2 constructs, all buffers used were from previously described methods (Mikel *et al.*, 2017). The filtered supernatant was mixed 1:1 with 2X binding buffer (100 mM NaH_2_PO_4_∙H_2_O pH 8.0, 30 mM imidazole, 600 mM NaCl) and loaded onto a 5 ml HiTrap TALON Crude column (GE Healthcare Life Sciences) with a 1 ml HiTrap Heparin HP column (GE Healthcare Life Sciences) in series via FPLC (ÄKTA Pure, GE Healthcare Life Sciences). The column was washed with 1X binding buffer (50 mM NaH_2_PO_4_∙H2O pH 8.0, 15 mM imidazole, 300 mM NaCl) and the protein eluted with elution buffer (50 mM NaH_2_PO_4_∙H_2_O pH 8.0, 200 mM imidazole, 300 mM NaCl) using an imidazole gradient from 15 mM to 200 mM. The protein was then buffer exchanged into STE buffer (10 mM Tris‐HCl pH 7.5, 1 mM EDTA, 100 mM NaCl) and flash frozen with liquid nitrogen before being stored at −80°C.

### Grid preparation and data collection

Quantifoil R1.2/1.3 300 mesh copper grids were plasma cleaned and coated with graphene oxide. Freshly purified MS2, produced with dimeric coat protein and maturation protein, was applied to grids at 4°C and >95% humidity, allowed to adsorb to the graphene oxide for 32 s before blotting for 2.5–3.5 s, and then plunge frozen in liquid ethane using the Vitrobot Mark IV (FEI). Two sets of datasets were collected in‐house on a Tecnai F20 (FEI) operated at 200 kV, using an early FEI Falcon detector, the first at a nominal magnification of ×100 k and acquired over an applied defocus range of −1.1 µm to −2.9 µm (comprising 1040 micrographs), and the second at ×150 k over an applied defocus range of −0.3 to −1.5 µm (comprising 1089 micrographs), both with a nominal dose of ~80 e/Å^2^.

### Image processing

Stacks were aligned and corrected for dose and beam‐induced motion using the program MotionCor2 (Zheng *et al.*, 2017), after which CTF estimation was performed using CTFFIND4 (Rohou and Grigorieff, 2015). For co‐processing of the x150k and x100k particles, the x150k images were down‐sampled in Fourier space to the same pixel size (1.0277 Å/pixel). Several rounds of manual particle selection using the program BOXER (Tang *et al.*, 2007) followed by 2D classification using RELION 2.1 (Scheres, 2012) were performed iteratively to obtain representative references for semi‐automatic particle selection. Single particle images were selected automatically using BATCHBOXER (Ludtke *et al.*, 1999) and subsequently used for classification and refinement.

Particles were subjected to two‐dimensional classification in 100 classes for each dataset and the icosahedral *T* = 3 and *T* = 4 particles selected for further processing based on clear convergence of these classes. There were sufficient particles to reach high resolution from the ×100 k dataset alone in the case of the *T* = 3 data (47 072 particles), whereas the *T* = 4 reconstruction required all particles from both datasets (120 804 particles) to reach a readily interpretable resolution because fewer particles were obtained. The selected particles were refined in a ‘gold‐standard’, independent half‐set, refinement, with a mask restricting the refinement to the capsid, although packaged RNA was weakly visible in unmasked reconstructions. The *T* = 3 refinement reached a resolution of 4 Å (8 993 particles, FSC = 0.143) within a tight mask, whereas the *T* = 4 refinement reached a slightly lower resolution of 6 Å (3 499 particles, FSC = 0.143) within a tight mask.

### Accession numbers

The *T* = 3 capsid reconstruction and PDB were deposited with the EMDB and PDB under codes EMD‐4989 and 6RRS respectively. The *T* = 4 capsid reconstruction and PDB were similarly deposited as EMD‐4990 and 6RRT respectively.

## Author contributions

MAC performed sample expression and purification. KR, NDMG, PAS and CHSA performed electron microscopy. KR, NDMG and CHSA analysed and interpreted structural data. NDMG, MAC and KR wrote the manuscript. CHSA and PSF conceived of the study.

## References

[mmi14406-bib-0001] Asensio, M.A. , Morella, N.M. , Jakobson, C.M. , Hartman, E.C. , Glasgow, J.E. , Sankaran, B. , *et al* (2016). A selection for assembly reveals that a single amino acid mutant of the bacteriophage MS2 coat protein forms a smaller virus‐like particle. Nano Letters, 16(9), 5944–5950. 10.1021/acs.nanolett.6b02948. 27549001

[mmi14406-bib-0002] Ashley, C.E. , Carnes, E.C. , Phillips, G.K. , Durfee, P.N. , Buley, M.D. , Lino, C.A. , *et al* (2011) Cell‐specific delivery of diverse cargos by bacteriophage MS2 virus‐like particles. ACS Nano, 5(7), 5729–5745. 10.1021/nn201397z. 21615170 PMC3144304

[mmi14406-bib-0003] Bazinet, C. and King, J. (1985) The DNA translocating vertex of dsDNA bacteriphage. Annual Review of Microbiology, 39(1), 109–138. 10.1146/annurev.mi.39.100185.000545. 2932996

[mmi14406-bib-0004] Brinton, C.C. , Gemski, P. and Carnahan, J . (2006). A new type of bacterial pilus genetically controlled by the fertility factor of *E. coli* K12 and its role in chromosome transfer. Proceedings of the National Academy of Sciences, 52(3), 776–783. 10.1073/pnas.52.3.776. PMC300345 14212557

[mmi14406-bib-0005] Caspar, D.L. and Klug, A. (1962) Physical principles in the construction of regular viruses. Cold Spring Harbor Symposia on Quantitative Biology, 27, 1–24. 10.1101/SQB.1962.027.001.005. 14019094

[mmi14406-bib-0006] Crick, F.H.C. and Watson, J.D. (1956) Structure of small viruses. Nature, 177(4506), 473–475. 13309339 10.1038/177473a0

[mmi14406-bib-0007] Curtiss, L.K. and Krueger, R.G. (1974) Localization of coliphage MS2 A‐protein. Journal of Virology, 14(3), 503–508. 4853028 10.1128/jvi.14.3.503-508.1974 PMC355543

[mmi14406-bib-0008] Dai, X. , Li, Z. , Lai, M. , Shu, S. , Du, Y. , Hong Zhou, Z. , *et al* (2017) In situ structures of the genome and genome‐delivery apparatus in a single‐stranded RNA virus. Nature, 541(7635), 112–116. 10.1038/nature20589. 27992877 PMC5701785

[mmi14406-bib-0009] Dent, K.C. , Thompson, R. , Barker, A.M. , Hiscox, J.A. , Barr, J.N. , Stockley, P.G. , *et al* (2013) The asymmetric structure of an icosahedral virus bound to its receptor suggests a mechanism for genome release. Structure, 21(7), 1225–1234. 10.1016/J.STR.2013.05.012. 23810697 PMC3701328

[mmi14406-bib-0010] Dykeman, E.C. , Stockley, P.G. and Twarock, R. (2010) Dynamic allostery controls coat protein conformer switching during MS2 phage assembly. Journal of Molecular Biology, 395(5), 916–923. 10.1016/J.JMB.2009.11.016. 19913554

[mmi14406-bib-0011] Fiers, W. , Contreras, R. , Duerinck, F. , Haegeman, G. , Iserentant, D. , Merregaert, J. , *et al* (1970) Complete nucleotide sequence of bacteriophage MS2 RNA: primary and secondary structure of the replicase gene. Nature, 260, 500–507. 10.1038/260500a0 1264203

[mmi14406-bib-0012] Golmohammadi, R. , Valegård, K. , Fridborg, K. and Liljas, L. (1993) The Refined Structure of Bacteriophage MS2 at 2·8 Å Resolution. Journal of Molecular Biology, 234(3), 620–639. 10.1006/JMBI.1993.1616. 8254664

[mmi14406-bib-0013] Gorzelnik, K.V. , Cui, Z. , Reed, C.A. , Jakana, J. , Young, R. and Zhang, J. (2016) Asymmetric cryo‐EM structure of the canonical Allolevivirus Qβ reveals a single maturation protein and the genomic ssRNA in situ. Proceedings of the National Academy of Sciences of the United States of America, 113(41), 11519–11524. 10.1073/pnas.1609482113. 27671640 PMC5068298

[mmi14406-bib-0014] Jung, J. , Grant, T. , Thomas, D.R. , Diehnelt, C.W. , Grigorieff, N. and Joshua‐Tor, L. (2019) High‐resolution cryo‐EM structures of outbreak strain human norovirus shells reveal size variations. Proceedings of the National Academy of Sciences, 116(26), 12828–12832. 10.1073/pnas.1903562116. PMC6601263 31182604

[mmi14406-bib-0015] Klug, A . (1999). The tobacco mosaic virus particle: structure and assembly. Philosophical Transactions of the Royal Society of London. Series B, Biological Sciences, 354(1383), 531–535. 10212932 10.1098/rstb.1999.0404 PMC1692534

[mmi14406-bib-0016] Knopp, Y. , Geis, F.K. , Heckl, D. , Horn, S. , Neumann, T. , Kuehle, J. , *et al* (2018) Transient retrovirus‐based CRISPR/Cas9 all‐in‐one particles for efficient, targeted gene knockout. Molecular Therapy ‐ Nucleic Acids, 13, 256–274. 10.1016/j.omtn.2018.09.006. 30317165 PMC6187057

[mmi14406-bib-0017] Koning, R.I. , Gomez‐Blanco, J. , Akopjana, I. , Vargas, J. , Kazaks, A. , Tars, K. , *et al* (2016) Asymmetric cryo‐EM reconstruction of phage MS2 reveals genome structure in situ. Nature Communications, 7(1), 12524 10.1038/ncomms12524. PMC5007439 27561669

[mmi14406-bib-0018] Krahn, P.M. , O'Callaghan, R.J. and Paranchych, W. (1972) Stages in phage R17 infection. VI. Injection of a protein and RNA into the host cell. Virology, 47(3), 628–637. 10.1016/0042-6822(72)90552-1. 4551992

[mmi14406-bib-0019] Legendre, D. and Fastrez, J. (2005) Production in *Saccharomyces cerevisiae* of MS2 virus‐like particles packaging functional heterologous mRNAs. Journal of Biotechnology, 117(2), 183–194. 10.1016/J.JBIOTEC.2005.01.010. 15823407

[mmi14406-bib-0020] Li, J. , Sun, Y. , Jia, T. , Zhang, R. , Zhang, K. and Wang, L. (2014) Messenger RNA vaccine based on recombinant MS2 virus‐like particles against prostate cancer. International Journal of Cancer, 134(7), 1683–1694. 10.1002/ijc.28482. 24105486

[mmi14406-bib-0021] Ludtke, S.J. , Baldwin, P.R. and Chiu, W. (1999) EMAN: semiautomated software for high‐resolution single‐particle reconstructions. Journal of Structural Biology, 128(1), 82–97. 10.1006/JSBI.1999.4174. 10600563

[mmi14406-bib-0022] Mastico, R.A. , Talbot, S.J. and Stockley, P.G. (1993) Multiple presentation of foreign peptides on the surface of an RNA‐free spherical bacteriophage capsid. Journal of General Virology, 74(4), 541–548. 10.1099/0022-1317-74-4-541. 7682249

[mmi14406-bib-0023] Meng, R. , Jiang, M. , Cui, Z. , Chang, J.‐Y. , Yang, K. , Jakana, J. , *et al* (2019) Structural basis for the adsorption of a single‐stranded RNA bacteriophage. Nature Communications, 10, 3130. 10.1038/s41467-019-11126-8. PMC6635492 31311931

[mmi14406-bib-0024] Mikel, P. , Vasickova, P. and Kralik, P. (2017) One‐plasmid double‐expression His‐tag system for rapid production and easy purification of MS2 phage‐like particles. Scientific Reports, 7(1), 17501 10.1038/s41598-017-17951-5. 29235545 PMC5727534

[mmi14406-bib-0025] Pasloske, B.L. , Walkerpeach, C.R. , Obermoeller, R.D. , Winkler, M. and DuBois, D.B. (1998) Armored RNA technology for production of ribonuclease‐resistant viral RNA controls and standards. Journal of Clinical Microbiology, 36(12), 3590–3594. 9817878 10.1128/jcm.36.12.3590-3594.1998 PMC105245

[mmi14406-bib-0026] Peabody, D.S. and Chakerian, A. (1999) Asymmetric contributions to RNA binding by the Thr 45 residues of the MS2 coat protein dimer. The Journal of Biological Chemistry, 274, 25403–25410. 10464269 10.1074/jbc.274.36.25403

[mmi14406-bib-0027] Peabody, D.S. and Lim, F. (1996) Complementation of RNA binding site mutations in MS2 coat protein heterodimers. Nucleic Acids Research, 24(12), 2352–2359. 10.1093/nar/24.12.2352. 8710507 PMC145953

[mmi14406-bib-0028] Plevka, P. , Tars, K. and Liljas, L. (2008) Crystal packing of a bacteriophage MS2 coat protein mutant corresponds to octahedral particles. Protein Science, 17, 1731–1739. 10.1110/ps.036905.108. 18662904 PMC2548359

[mmi14406-bib-0029] Plevka, P. , Tars, K. and Liljas, L. (2009) Structure and stability of icosahedral particles of a covalent coat protein dimer of bacteriophage MS2. Protein Science, 18, 1653–1661. 10.1002/pro.184. 19521994 PMC2776953

[mmi14406-bib-0030] Prasad, B.V.V. and Schmid, M.F . (2012).Principles of virus structural organizationIn: Rossmann M and Rao V (Eds.) Viral Molecular Machines. Advances in Experimental Medicine and Biology. Boston, MA: Springer, Vol. 726, pp. 17–47. 10.1007/978-1-4614-0980-9_3. PMC3767311 22297509

[mmi14406-bib-0031] Ran, F.A. , Cong, L. , Yan, W.X. , Scott, D.A. , Gootenberg, J.S. , Kriz, A.J. , *et al* (2015) In vivo genome editing using *Staphylococcus aureus* Cas9. Nature, 520(7546), 186–191. 10.1038/nature14299. 25830891 PMC4393360

[mmi14406-bib-0032] Roberts, J.W. and Steitz, J.E. (2006). The reconstitution of infective bacteriophage R17. Proceedings of the National Academy of Sciences, 58(4), 1416–1421. 10.1073/pnas.58.4.1416 PMC223940 5237875

[mmi14406-bib-0033] Rohou, A. and Grigorieff, N. (2015) CTFFIND4: fast and accurate defocus estimation from electron micrographs. Journal of Structural Biology, 192(2), 216–221. 10.1016/j.jsb.2015.08.008. 26278980 PMC6760662

[mmi14406-bib-0034] Rolfsson, Ó. , Toropova, K. , Ranson, N.A. and Stockley, P.G. (2010) Mutually‐induced conformational switching of RNA and coat protein underpins efficient assembly of a viral capsid. Journal of Molecular Biology, 401(2), 309–322. 10.1016/J.JMB.2010.05.058. 20684044 PMC4793595

[mmi14406-bib-0035] Scheres, S.H.W. (2012) RELION: Implementation of a Bayesian approach to cryo‐EM structure determination. Journal of Structural Biology, 180(3), 519–530. 10.1016/j.jsb.2012.09.006. 23000701 PMC3690530

[mmi14406-bib-0036] Stockley, P.G. , Rolfsson, O. , Thompson, G.S. , Basnak, G. , Francese, S. , Stonehouse, N.J. , *et al* (2007) A simple, RNA‐mediated allosteric switch controls the pathway to formation of a T = 3 viral capsid. Journal of Molecular Biology, 369(2), 541–552. 10.1016/J.JMB.2007.03.020. 17434527 PMC7612263

[mmi14406-bib-0037] Stockley, P.G. , Ranson, N.A. and Twarock, R. (2013) A new paradigm for the roles of the genome in ssRNA viruses. Future Virology, 8(6), 531–543. 10.2217/fvl.12.84.

[mmi14406-bib-0038] Stockley, P.G. , White, S.J. , Dykeman, E. , Manfield, I. , Rolfsson, O. , Patel, N. , *et al* (2016) Bacteriophage MS2 genomic RNA encodes an assembly instruction manual for its capsid. Bacteriophage, 6(1), e1157666 10.1016/j.jmb.2015.11.014. 27144089 PMC4836477

[mmi14406-bib-0039] Sugiyama, T. , Hebert, R.R. and Hartman, K.A. (1967) Ribonucleoprotein complexes formed between bacteriophage MS2 RNA and MS2 protein in vitro. Journal of Molecular Biology, 25(3), 455–463. 10.1016/0022-2836(67)90198-2. 6035286

[mmi14406-bib-0040] Sun, S. , Rao, V.B. and Rossmann, M.G. (2010) Genome packaging in viruses. Current Opinion in Structural Biology, 20(1), 114–120. 10.1016/j.sbi.2009.12.006. 20060706 PMC2948483

[mmi14406-bib-0041] Tang, G. , Peng, L. , Baldwin, P.R. , Mann, D.S. , Jiang, W. , Rees, I. , *et al* (2007) EMAN2: an extensible image processing suite for electron microscopy. Journal of Structural Biology, 157(1), 38–46. 10.1016/j.jsb.2006.05.009. 16859925

[mmi14406-bib-0042] Toropova, K. , Basnak, G. , Twarock, R. , Stockley, P.G. and Ranson, N.A. (2008) The three‐dimensional structure of genomic RNA in bacteriophage MS2: implications for assembly. Journal of Molecular Biology, 375(3), 824–836. 10.1016/J.JMB.2007.08.067. 18048058

[mmi14406-bib-0043] Uhlenbeck, O.C. (1998) A coat for all sequences. Nature Structural Biology, 5(3), 174–176. 10.1038/nsb0398-174. 9501905

[mmi14406-bib-0044] Valegard, K. , Liljas, L. , Fridborg, K. and Unge, T. (1990) The three‐dimensional structure of the bacterial virus MS2. Nature, 345, 36–41. 2330049 10.1038/345036a0

[mmi14406-bib-0045] Valentine, R.C. and Strand, M. (1965) Complexes of F‐pili and RNA bacteriophage. Science, 148(3669), 511–513. 10.1126/science.148.3669.511. 14263773

[mmi14406-bib-0046] Venkatakrishnan, B. and Zlotnick, A. (2016) The structural biology of hepatitis B virus: form and function. Annual Review of Virology, 3(1), 429–451. 10.1146/annurev-virology-110615-042238. PMC5646271 27482896

[mmi14406-bib-0047] Wei, B. , Wei, Y. , Zhang, K. , Yang, C. , Wang, J. , Xu, R. , *et al* (2008) Construction of armored RNA containing long‐size chimeric RNA by increasing the number and affinity of the pac site in exogenous RNA and sequence coding coat protein of the MS2 bacteriophage. Intervirology, 51(2), 144–150. 10.1159/000141707. 18594159 PMC7179527

[mmi14406-bib-0048] Wu, M. , Sherwin, T. , Brown, W.L. and Stockley, P.G. (2005) Delivery of antisense oligonucleotides to leukemia cells by RNA bacteriophage capsids. Nanomedicine: Nanotechnology, Biology, and Medicine, 1(1), 67–76. 10.1016/j.nano.2004.11.011. 17292060

[mmi14406-bib-0049] Zhan, S. , Li, J. , Xu, R. , Wang, L. , Zhang, K. and Zhang, R. (2009) Armored long RNA controls or standards for branched DNA assay for detection of human immunodeficiency virus type 1. Journal of Clinical Microbiology, 47(8), 2571–2576. 10.1128/JCM.00232-09. 19494069 PMC2725685

[mmi14406-bib-0050] Zhang, L. , Sun, Y. , Chang, L. , Jia, T. , Wang, G. , Zhang, R. , *et al* (2015) A novel method to produce armored double‐stranded DNA by encapsulation of MS2 viral capsids. Applied Microbiology and Biotechnology, 99(17), 7047–7057. 10.1007/s00253-015-6664-4. 25981999 PMC7079959

[mmi14406-bib-0051] Zhao, L. , Kopylov, M. , Potter, C.S. , Carragher, B. and Finn, M.G. (2019) Engineering the PP7 virus capsid as a peptide display platform. ACS Nano, 13(4), 4443–4454. 10.1021/acsnano.8b09683. 30912918 PMC6991139

[mmi14406-bib-0052] Zheng, S.Q. , Palovcak, E. , Armache, J.P. , Verba, K.A. , Cheng, Y. , *et al* (2017) MotionCor2: Anisotropic correction of beam‐induced motion for improved cryo‐electron microscopy. Nature Methods, 14(4), 331–332. 10.1038/nmeth.4193. 28250466 PMC5494038

