# Supplementary material for: Bacteriophage MS2 displays unreported capsid variability assembling T = 4 and mixed capsids
Source: Mol Microbiol. 2019 Nov 5;113(1):143–52. doi: 10.1111/mmi.14406 (PMC7027807; doi:10.1111/mmi.14406)
Supplement: Supplementary file 1 [file MMI-113-143-s001.pdf]

Supplementary Information:

Bacteriophage MS2 displays unreported capsid variability assembling *T*=4 and mixed capsids

T=4 capsids observed for MS2 virus-like particles

[1*]Natàlia de Martín Garrido, [1,2*]Michael A. Crone, [1]Kailash Ramlaul, [3]Paul A. Simpson, [1,2,4†]Paul S. Freemont & [1†]Christopher H. S. Aylett

[1]Section of Structural and Synthetic Biology, Department of Infectious Disease, Imperial College London, London, SW7 2AZ

[2]UK DRI Care Research and Technology Centre at Imperial College London

[3]Centre for Structural Biology, Department of Life Sciences, Imperial College London, London, SW7 2AZ

[4]London BioFoundry, Imperial College Translation & Innovation Hub, White City Campus, 80 Wood Lane, London, W12 0BZ

Keywords*:* Phage MS2; Capsid; Maturation protein; Triangulation number; Virion

[*] Natàlia de Martín Garrido and Michael A. Crone should be considered joint first author.

[†] To whom correspondence may be addressed:

CHSA: c.aylett@imperial.ac.uk

PSF: p.freemont@imperial.ac.uk

Supplementary figures

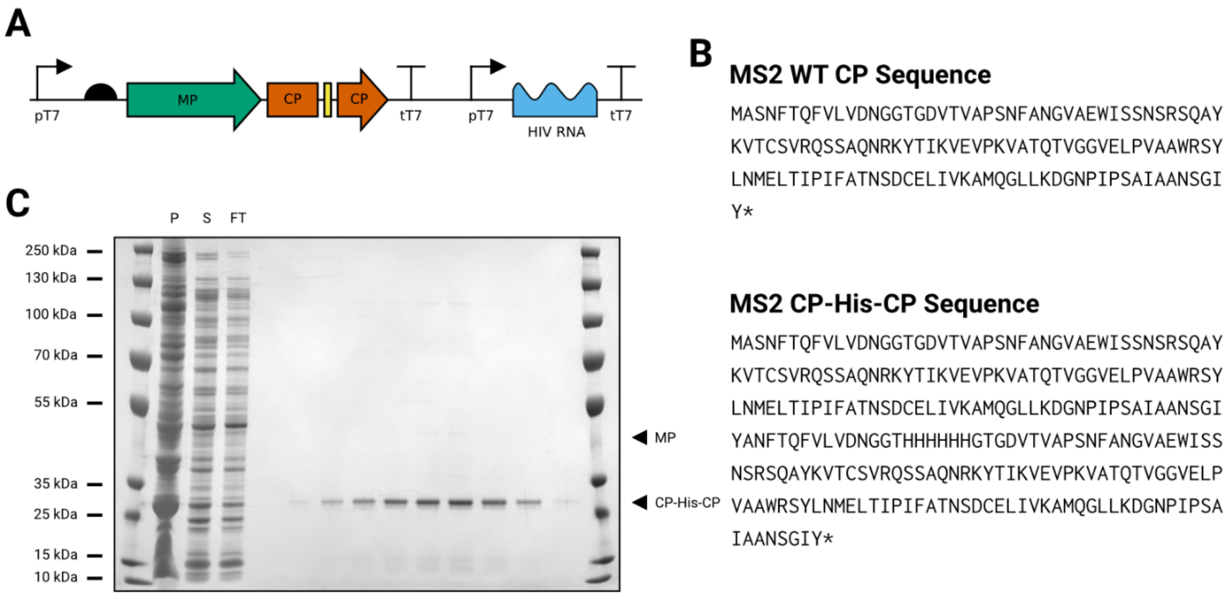

Supplementary Figure 1: MS2 CP-His-CP genetic construct and purification of MS2 His-tagged construct. A) The genetic construct (Der et al., 2017) of the MS2 CP-His-CP expression vector (pMC042). The maturation protein, coat protein dimer and user-specified RNA are coexpressed under T7 promoters (pT7). The MS2 coat protein subunits (orange) are linked with a His-tag (yellow), which allows for affinity purification. The RNA to be packaged is a non-coding RNA sequence (blue) with accompanying *pac site* (Supplementary Fig. 2A). B) Sequences of both the wild type (top) and the CP-His-CP MS2 dimeric constructs (bottom). C) SDS-PAGE gel of expressed and purified MS2 phage-like particles: P correspond to the cell pellet, S to the soluble fraction after cell lysis, FT to the flow through after running the sample on a HiTrap TALON crude column with a HiTrap Heparin HP column in series, and the remaining lanes correspond to elution fractions. The CP-His-CP dimer (~28 kDa) is clearly visible and the Maturation protein (~44 kDa) is barely visible but was confirmed using Mass Spectrometry (Supplementary Table 1). Eluted fractions were then collected, concentrated and buffer exchanged into STE buffer.

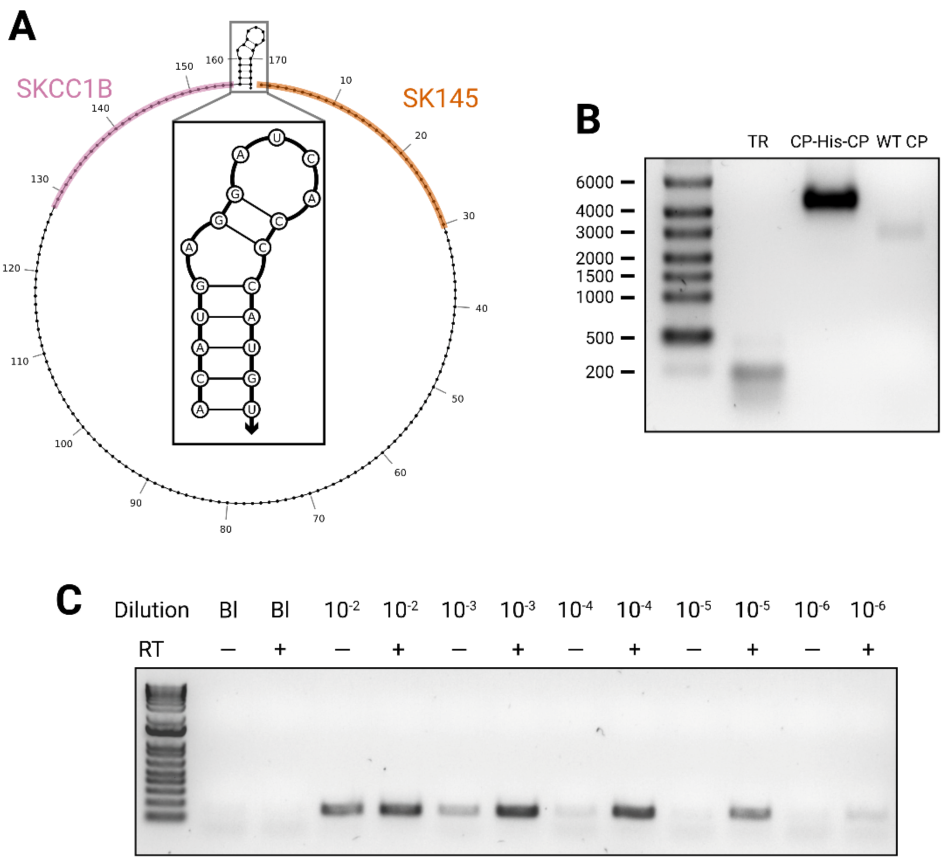

Supplementary Figure 2: Diagrammatic representation of the heterologous RNA, Electrophoretic Mobility Shift Assay and RT-PCR lysed MS2 virus-like particles. A) Representation of the packaged RNA sequence, showing the location of the two RT-PCR primers (SK145 and SKCC1B) and the secondary structure of the 3' *pac site* (inset). B) Electrophoretic Mobility Shift Assay of unpackaged transcribed RNA, RNA packaged in the CP-His-CP MS2 VLP and RNA packaged in the wild type CP MS2 VLP. Packaged RNA migrates more slowly through the agarose gel. On initial analysis the CP-His-CP MS2 construct appeared to be more stable and it was used for further cryo-EM analysis. C) RT-PCR of the CP-His-CP VLP packaged RNA using SK145 and SKCC1B primers (Supplementary Fig. 2A, Supplementary Table 3). Residual plasmid DNA is present in the purified sample at low concentrations even after the addition of nucleases. Reactions were therefore run with (+) and without (-) Reverse Transcriptase (RT) with a blank sample (Bl) and with serial dilutions of the VLPs. At dilutions below 10^{-4} the presence of only the packaged RNA is confirmed.

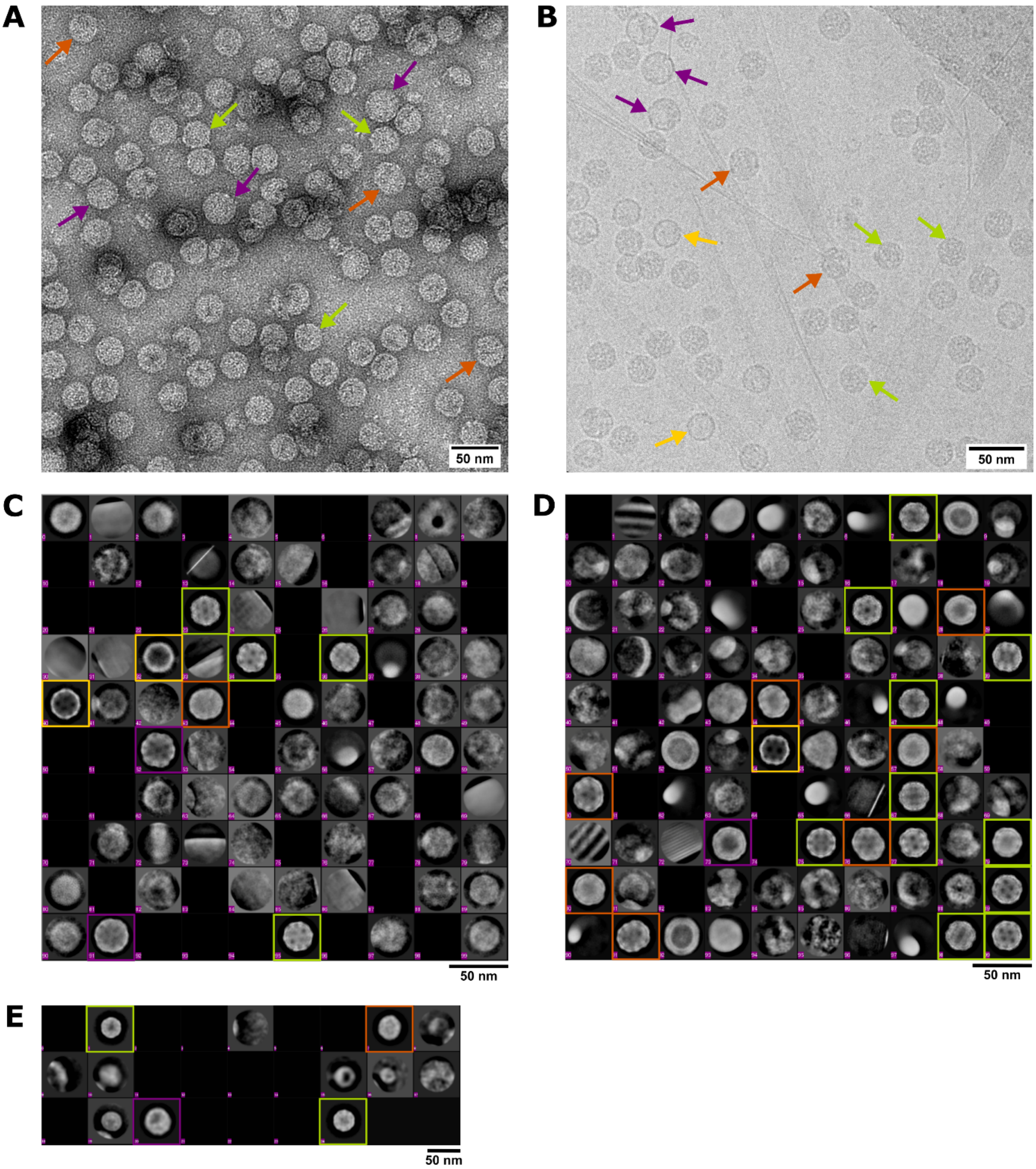

Supplementary Figure 3: Cryo-EM studies of MS2 capsid packed with an exogeneous RNA. A) A representative micrograph of the negatively stained grids of purified CP-His-CP construct showing VLP assembly and heterogeneity in their capsid size: green arrows point at “small” particles, purple at “large” particles and orange at “non-spherical” particles. B) A sample micrograph of the cryo-grids prepared with the purified CP-His-CP MS2 dimer packed with the 155bp RNA. Arrows point at different sizes and shapes of particles as in

Supplementary Fig. 3A with additional yellow arrows pointing at empty capsids. C) 2D classes showing the different capsid types observed in cryo-EM micrographs of purified CP-His-CP MS2 VLPs. Colour code is the same as in Supplementary Fig. 3B D) 2D classes showing that particles were classified similarly when VLPs are assembled with the wild-type construct, containing monomeric CP and MP coexpressed with the 155bp RNA. Colour code is the same as in Supplementary Fig. 3B. E) 2D classification of negatively stained grids of wild-type construct coexpressed with 155 bp RNA but without MP. Colour code is the same as in Supplementary Fig. 3A.

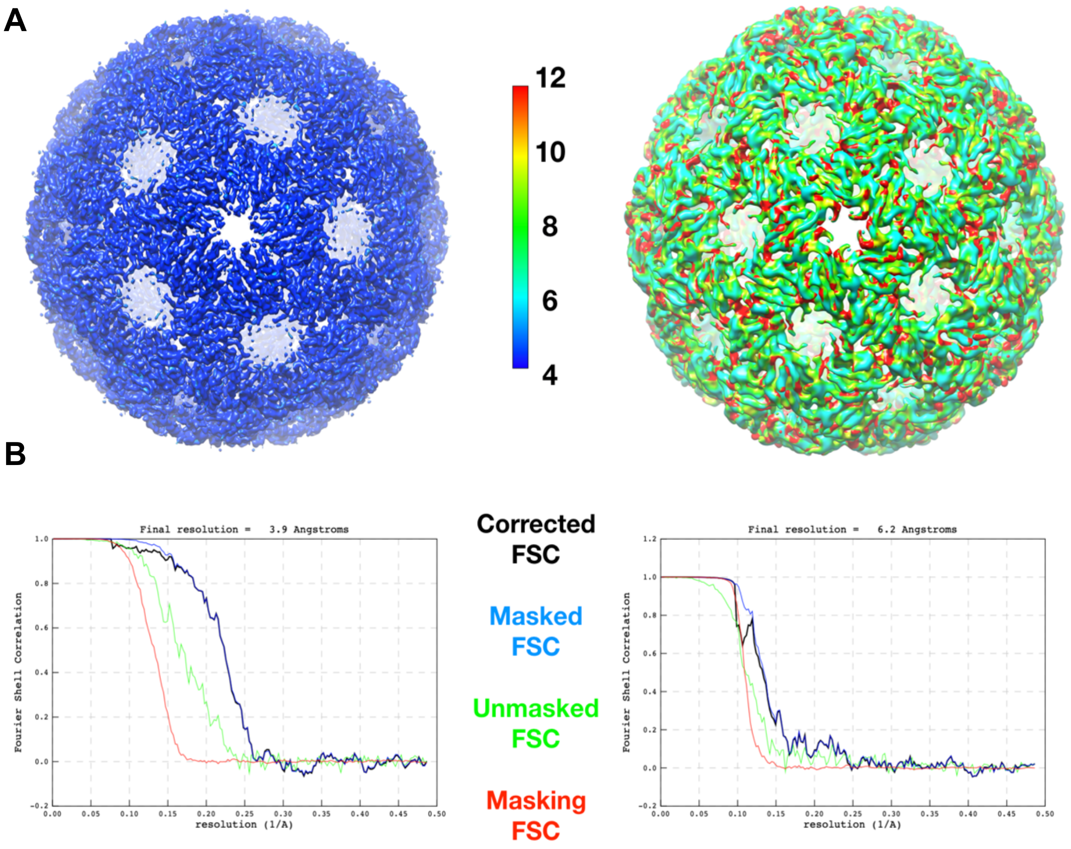

Supplementary Figure 4: Cryo-EM three-dimensional reconstruction of *T*=3 and *T*=4 MS2 capsids. A) Local resolution map from Relion for the T=3 (left) and T=4 (right) MS2 capsid forms. B) FSC curve output from Relion for the T=3 (left) and T=4 (right) reconstructions.

Supplementary tables

Observed m/z	Mr (expt)	Mr (calc)	Mass Difference	Peptide Score	Peptide Sequence
837.8644	1673.7142	1673.7421	-0.0278	77.93	(R)VYADGETEDNSFSLK(Y)
705.3562	1408.6979	1408.7198	-0.0219	57.08	(R)GALSVTSIDQGAYK(R)
520.7539	1039.4932	1039.5087	-0.0155	52.4	(K)AGFGFSLDAR(S)
438.239	874.4634	874.476	-0.0125	49.41	(R)ASTEVLQK(V)
830.4312	1658.8479	1659.871	-1.0231	71.71	(K)VTQGNFNLGVALAEAR(S)
553.9566	1658.8481	1659.871	-1.0229	54.93	(K)VTQGNFNLGVALAEAR(S)
561.2836	1680.8291	1681.89	-1.0609	51.41	(K)VTQGNFNLGVALAEAR(S)
766.4269	1530.8392	1530.8617	-0.0225	94.06	(R)STASQLATQTIALVK(A)
497.2462	992.4779	992.4927	-0.0147	49.11	(R)YLALNEDR(K)
596.3123	1785.915	1785.9447	-0.0298	58.67	(K)SPFSMVHTLDALALIR(Q)

MRAFSTLDRE NETFVPSVRV YADGETEDNS FSLKYRSNWT PGRFNSTGAK TKQWHYPSPY	60
SRGALSVTSI DQGAYKRSGS SWGRPYEEKA GFGFSLDARS CYSLFPVSQN LTYIEVPQNV	120
ANRASTEVLQ KVTQGNFNLG VALAEARSTA SQLATQTIAL VKAYTAARRG NWRQALRYLA	180
LNEDRKFRSK HVAGRWLELQ FGWLPLMSDI QGAYEMLTKV HLQEFLPMRA VRQVGTNIKL	240
DGRLSYPAAN FQTTCNISRR IVIWFYINDA RLAWLSSLGI LNPLGIVWEK VPFSFVVDWL	300
LPVGNMLEGL TAPVGCSYMS GTVTDVITGE SIISVDAPYG WTVERQGTAK AQISAMHRGV	360
QSVWPTTGAY VKSPFSMVHT LDALALIRQR LSR	393

Supplementary Table 1. Peptide Fingerprint of the MS2 Maturation Protein. Mass spectrometry showing the significant peptides detected, other than those for porcine trypsin. The peptides showed 25% coverage (highlighted in grey) for the MS2 Maturation Protein with a Mascot score of 448. This rose to 46% and 799 when including non-significant peptide matches.

pMC036	https://benchling.com/mcroneicl/f/JHdCLGiL-plasmids/seq-etW0SrXm-pmc036-ms2_plp/edit
pMC037	https://benchling.com/mcroneicl/f/JHdCLGiL-plasmids/seq-KRAmpE3Q-pmc037-hisms2_plp_pac/edit
pMC042	https://benchling.com/mcroneicl/f/JHdCLGiL-plasmids/seq-CLPdlWiQ-pmc042-hisms2_plp_pac_h1/edit
pMC046	https://benchling.com/mcroneicl/f/JHdCLGiL-plasmids/seq-GVbF8UQh-pmc046-wtms2_plp_pac_h1_cole1/edit
pMC054	https://benchling.com/mcroneicl/f/JHdCLGiL-plasmids/seq-DvwftFKS-pmc054-wtms2_nomat_pac_h1_cole1/edit

Supplementary Table 2: List of plasmids used for hecloning.

Name	Sequence
HIV Packaged Sequence	AGTGGGGGGACATCAAGCAGCCATGCAAATGTTAAAAGATACCATCAATGAGGAGGC TGCAGAATGGGATAGATTACATCCAGTACATGCAGGGCCTATTGCACCAGGCCAAAT GAGAGAACCAAGGGGAAGTGACATAGCAGGAACTACTAGTA
**Primer Name**	**Primer Sequence**
SK145	AGTGGGGGGACATCAAGCAGCCATGCAAAT
SKCC1B	TACTAGTAGTTCCTGCTATGTCACTTCC
priDMC022	GTATAATACGACTCACTATAGG
priDMC078	GGTGGTCTCGGACAAAAAACCCCTCAAGACCC
priDMC089	TTACAAAAAACCCCTCAAGACCCGTTTAG
priDMC090	GCCTCTAAACGGGTCTTGAGGGGTTTTTT
priDMC091	AGGCCCCAAGGGGTTATGCTAGTTATTGCTCAGC
priDMC092	CACCGCTGAGCAATAACTAGCATAACCCCTTGGG
priDMC094	GTGGGTCTCGTGGCTATCGCTGTAGGTAGC
priDMC095	GTGGGTCTCCGCCAATTTAAATCTCCTTCTTAAAGTTAAACA
priDMC096	GTGGGTCTCCGAGGTAATACGACTCACTATAGGGGAA
priDMC110	CTGGTCTCTCCTCACTGGCCGTCGTTTTACAAC
priDMC111	CTGGTCTCTGTAATTTCACTGCCCGCTTTCC
priDMC119	TACTAGTAGTTCCTGCTATGTCACTTCCCCTTGGTTCTCTCATTTGGCCTGGTGCAATA GG CCCTGCATGTACTGGATGTAATCTATCCCATTCTGCAGCCTCCTCATTGATGGTATCTT TT AACATTTGCATGGCTGCTTGATGTCCCCCCACTCCTATAGTGAGTCGTATTATAC
priDMC122	ATGTTACTAGTAGTTCCTGCTATGTCACTTCCCCT
priDMC123	ACCAAGGGGAAGTGACATAGCAGGAACTACTAGTA
priDMC124	TGGTTCTCTCATTTGGCCTGGTGCAATAGGCC
priDMC125	GCAGGGCCTATTGCACCAGGCCAAATGAGAGA
priDMC126	CTGCATGTACTGGATGTAATCTATCCCATTCTGCAGCCTCCTCATT
priDMC127	CATCAATGAGGAGGCTGCAGAATGGGATAGATTACATCCAGTACAT
priDMC128	GATGGTATCTTTTAACATTTGCATGGCTGCTTGATGTCCCCCCACTC
priDMC129	ATAGGAGTGGGGGGACATCAAGCAGCCATGCAAATGTTAAAAGATAC
priDMC148	CATGGTCTCTAGCCTGTGAACGCGAGTTAG

priDMC149	CAGGGTCTCTGGCTTACAAAGTAACCTGTAGCGTTC
priDMC178	GTAGGTCTCCTGTCCTTCTAGTGTAGCCGTAGTTAGGC
priDMC179	GTAGGTCTCCGACAGTATTTGGTATCTGCGCTCTG
priDMC271	CTGGGTCTCCATAGCCCTCAACCGGAGTTTG
priDMC272	CTGGGTCTCCCTATTTAAATCTCCTTCTTAAAGTTAAACAAAATTATTTG

Supplementary Table 3: Nucleotide sequence of the 155bp RNA packed on MS2 capsids and list of primers used for cloning.

Supplementary Methods

Cloning

All cloning was done using 'Golden Gate' assembly. All Golden Gate assembly reactions were incubated at either 37°C for 18 hours or using cycling conditions described previously (Engler, Kandzia, Marillonnet, Eldik, & Botterman, 2008). All phosphorylations were performed in ligase reaction buffer (Promega). All PCR amplification reactions were performed using Q5® High-Fidelity DNA Polymerase (NEB) and were subsequently DpnI (NEB) digested and gel purified (Zymoclean Gel DNA Recovery Kit, Zymo Research). All plasmids were sequence verified (Eurofins Genomics).

A previously described double-expression construct with a CP-His-CP dimer (Mikel, Vasickova, & Kralik, 2017) as ordered in a cloning vector from GeneArt (Thermo Fisher Scientific). A T7 promoter and T7 terminator were incorporated using 'Golden Gate' assembly. To insert the T7 promoter, the plasmid backbone from GeneArt was amplified using priDMC094 and priDMC110 and the T7 promoter and RBS were amplified from pET28a-MH6-RspCas13d using priDMC096 and priDMC095 (pET28a-MH6-RspCas13d was a gift from Arbor Biotechnologies (Addgene plasmid #108305)). A 'Golden Gate' reaction was setup with amplified T7 promoter (in excess), amplified backbone, ligase reaction buffer (Promega), 20U T4 DNA ligase (Promega) and 20U BsaI-HFv2 (NEB). The resultant plasmid was then amplified using priDMC094 and priDMC110. T7 terminator oligonucleotides (priDMC092 and priDMC091, priDMC090 and priDMC089) were phosphorylated with T4 PNK (Thermo Fisher Scientific), annealed and diluted 1:5. A Golden Gate reaction was setup with T7 terminator oligonucleotides (1 µl in 20 µl reaction), amplified backbone and other constituents described above. The resultant expression construct was named pMC036.

In order to package the CP-His-CP dimer with an RNA sequence, pMC036 was amplified using priDMC111 and priDMC078. A synthetic construct with a T7 promoter, Esp3I Type IIs cloning sites, a c-variant *pac site* (Wei et al., 2008) and T7 terminator were ordered as a gBlock (IDT). A Golden Gate reaction was setup with the synthetic construct, amplified

backbone and other constituents described above. The resultant construct was named pMC037 and allowed for the insertion of any RNA sequence for packaging using the Esp3I Type IIs cloning sites.

A 155 bp sequence from the HIV-1 *gag* gene (Supplementary Table 3) was selected for packaging (position 1359-1513 of reference strain HXB2 (accession number **K03455.1**), but the South African Subtype C consensus). A set of four oligos (priDMC129 and priDMC128, priDMC127 and priDMC126, priDMC125 and priDMC124, priDMC123 and priDMC122) were phosphorylated with T4 PNK (Thermo Fisher Scientific), annealed and diluted 1:5. A Golden Gate reaction was setup with the annealed, diluted HIV oligonucleotides (1µl of each in a 20µl reaction), pMC037, ligase reaction buffer (Promega), 20U T4 DNA ligase (Promega) and 10U Esp3I (Thermo Fisher Scientific). The plasmid was named pMC042.

A plasmid with the wild type MS2 coat protein was made by amplifying pMC042 with primers priDMC149 and priDMC148. A Golden Gate reaction was then performed with the amplified product and BsaI (as described above). The resultant construct did not perform well on preliminary protein expression experiments and therefore a lower copy number plasmid was created by introducing an A>G mutation in the origin of replication. This was performed by amplifying the above construct with priDMC179 and priDMC178 and performing a Golden Gate reaction as described above with BsaI. The resultant construct was named pMC046.

A plasmid with the wild type MS2 and without the maturation protein was created by amplifying the pMC046 backbone with priDMC271 and priDMC272. A Golden Gate reaction with BsaI was then performed to give pMC054.

pMC042 was used for all CP-His-CP experiments, pMC046 was used for all WT CP experiments and pMC054 was used to confirm that even without the maturation protein, particles still have both T=3 and T=4 architectures.

pMC037 and pMC051 (pMC037 with a lower copy origin) are available from Addgene.

Electrophoretic Mobility Shift Assay

In order to generate an unpackaged RNA control HIV sequence, DNA oligos were first annealed (priDMC119 and priDMC022) in Nuclease-Free Duplex Buffer (IDT). Annealed DNA (100 nM) was transcribed *in vitro* (TranscriptAid T7 High Yield Transcription Kit, ThermoFisher Scientific) and purified (RNA Clean and Concentrator Kit, Zymo Research). The assay was then run on a 1% agarose gel stained with SYBR Safe (ThermoFisher Scientific).

RT-PCR

Serial dilutions of prepared CP-His-CP MS2 VLPs were denatured at 65°C for 5 minutes. Reactions for each dilution were then prepared with and without M-MuLV Reverse Transcriptase (NEB) according to the manufacturer's instructions with the SKCC1B reverse primer. The reactions were then incubated at 42°C for one hour before being heat inactivated at 65°C for 20 minutes. PCR reactions using Q5® High-Fidelity DNA Polymerase (NEB) with the SK145 and SKCC1B primers were then setup with 1 µl template from the RT-PCR reaction. Resultant reactions were then run on a 1% agarose gel stained with SYBR Safe (ThermoFisher Scientific).

Mass Spectrometry

Samples were prepared as described previously (Shevchenko, Tomas, Havli, Olsen, & Mann, 2006). Extracted trypsin-digested products were then analysed using an auto-MS/MS method on a Q-ToF LC/MS system (Agilent). Output data was converted to a peak list using the Agilent Spectrum Mill MS Proteomics Workbench and Mascot (Perkins, Pappin, Creasy, & Cottrell, 1999) was used to query the SwissProt (2019_03) database (Bairoch & Apweiler, 1996). Significant identity matches ($p<0.05$) were selected and the Mascot score and protein coverage obtained.

Negative stain EM

Negatively stained grids were prepared using the carbon floating method (Harris, 1982). Sheets of mica with a thin deposited layer of amorphous carbon were floated in a reservoir of MS2 phage sample, before floating in a reservoir of 2% (w/v) uranyl acetate. The floated carbon was recovered onto a plasma-cleaned Quantifoil 1.2/1.3 300 mesh copper grids and left to air-dry. Grids were imaged with a Tecnai T12 electron microscope at 69 000x magnification.

References

Bairoch, A., & Apweiler, R. (1996). The SWISS-PROT protein sequence data bank and its new supplement TREMBL. *Nucleic Acids Research*, *24*(1), 21–25.

Der, B. S., Glassey, E., Bartley, B. A., Enghuus, C., Goodman, D. B., Gordon, D. B., … Gorochowski, T. E. (2017). DNAplotlib: Programmable Visualization of Genetic Designs and Associated Data. *ACS Synthetic Biology*, *6*(7), 1115–1119. https://doi.org/10.1021/acssynbio.6b00252

Engler, C., Kandzia, R., Marillonnet, S., Eldik, G. Van, & Botterman, J. (2008). A One Pot, One Step, Precision Cloning Method with High Throughput Capability. *PLoS ONE*, *3*(11), e3647. https://doi.org/10.1371/journal.pone.0003647

Harris, J. R. (1982). The production of paracrystalline two-dimensional monolayers of purified protein molecules. *Micron (1969)*, *13*(2), 147–168. https://doi.org/10.1016/0047-7206(82)90081-4

Mikel, P., Vasickova, P., & Kralik, P. (2017). One-plasmid double-expression His-tag system for rapid production and easy purification of MS2 phage-like particles. *Scientific Reports*, *7*(1), 17501. https://doi.org/10.1038/s41598-017-17951-5

Perkins, D. N., Pappin, D. J. C., Creasy, D. M., & Cottrell, J. S. (1999). Probability-based protein identification by searching sequence databases using mass spectrometry data. *Electrophoresis*, *20*(18), 3551–3567. https://doi.org/10.1002/(SICI)1522-2683(19991201)20:18<3551::AID-ELPS3551>3.0.CO;2-2

Shevchenko, A., Tomas, H., Havli, J., Olsen, J. V, & Mann, M. (2006). In-gel digestion for mass spectrometric characterization of proteins and proteomes. *Nature Protocols*, *1*(6), 2856–2860. https://doi.org/10.1038/nprot.2006.468

Wei, B., Wei, Y., Zhang, K., Yang, C., Wang, J., Xu, R., … Li, J. (2008). Construction of Armored RNA Containing Long-Size Chimeric RNA by Increasing the Number and Affinity of the Pac Site in Exogenous RNA and Sequence Coding Coat Protein of the MS2 Bacteriophage. *Intervirology*, *51*(2), 144–150. https://doi.org/10.1159/000141707
